# Impact of the Sediment Organic vs. Mineral Content on Distribution of the Per- and Polyfluoroalkyl Substances (PFAS) in Lake Sediment

**DOI:** 10.3390/ijerph17165642

**Published:** 2020-08-05

**Authors:** Dauren Mussabek, Kenneth M. Persson, Ronny Berndtsson, Lutz Ahrens, Kei Nakagawa, Tomomi Imura

**Affiliations:** 1 Division of Water Resources Engineering, LTH Lund University, 22100 Lund, Sweden; kenneth_m.persson@tvrl.lth.se (K.M.P.); Ronny.Berndtsson@tvrl.lth.se (R.B.); 2 Department of Aquatic Sciences and Assessment, Swedish University of Agricultural Sciences (SLU), 75007 Uppsala, Sweden; lutz.ahrens@slu.se; 3 Sweden Water Research AB, 22370 Lund, Sweden; 4 South Sweden Water Supply AB (Sydvatten AB), 21532 Malmö, Sweden; 5 Center for Middle Eastern Studies (CMES), Lund University, 22100 Lund, Sweden; 6 Institute of Integrated Sciences and Technology, Nagasaki University, Nagasaki 852-8521, Japan; kei-naka@nagasaki-u.ac.jp; 7 Faculty of Environmental Sciences, Nagasaki University, Nagasaki 852-8521, Japan; bb40116015@ms.nagasaki-u.ac.jp

**Keywords:** PFAS, AFFF, sediment, water, distribution

## Abstract

Contamination of the water and sediment with per- and polyfluoroalkyl substances (PFAS) was studied for the lake impacted by the release of PFAS-containing aqueous film forming foam (AFFF). PFAS concentrations were analyzed in lake water and sediment core samples. ΣPFAS concentrations were in the range of 95–100 ng L^−1^ in the lake water and 3.0–61 µg kg^−1^ dry weight (dw) in sediment core samples, both dominated by perfluorohexane sulfonate, perfluorooctane sulfonate; 6:2 fluortelomer sulfonate was inconsistently present in water and sediment core samples. The sediment–water partitioning coefficients (log *K_d_*) were estimated and ranged 0.6–2.3 L kg^−1^ for individual perfluoroalkyl carboxylates (PFCAs) and 0.9–5.6 L kg^−1^ for individual perfluoroalkane sulfonates (PFSAs). The influence of the sediment inorganic content and organic matter on PFAS distribution was investigated. In studied sediments, the mineral content (corresponding to <5% of the bulk media mass) was mainly represented by sulfur, iron and calcium. The PFAS distribution was found strongly connected to the sediment mineral content (i.e., Fe, Pb, Rb and As), whereas the sediment organic carbon content did not to have a direct influence on the PFAS distribution. The aim of this study was to improve our understanding of the PFAS distribution in the natural heterogeneous media.

## 1. Introduction

Per- and polyfluoroalkyl substances (PFAS) have frequently been in focus due to their persistent, bio-accumulative and potentially toxic characteristics [1,2]. The distribution of PFAS in the environment has mainly been associated with mobility and chemical stability of the substances in aquatic environment [3,4]. Important emission sources were often connected to use of the PFAS-containing aqueous film forming foam (AFFF) [4,5]. The AFFF application in extinguishing of hydrocarbon-fuel fires during training and emergency events can lead to severe contamination of the site surrounding environment [5,6].

The ubiquitous presence of PFAS in soil, water and biota represents a potential hazard to nature and public health [7,8,9]. Furthermore, the source water contamination can lead to continues human exposure via drinking water [10,11]. The population exposure and related health effects are yet subject to further investigation [12,13,14], not least concerning AFFF. In Sweden, approximately one-third of the airfields were connected to the source water contamination due to historical AFFF emission [15,16]. In present study, the investigated area represents the case of severe drinking water contamination, with over 16,000 individuals exposed among the population between the 1980s and 2013 [17,18]. The health-related risks have been under extensive investigation, including the assessment of exposure conditions and associated PFAS levels in human serum [13,19]. A better understanding of the PFAS distribution in the area was needed.

The retrospective estimates on contamination levels in drinking water are important for the human exposure investigation, in particular for the dose–response relationships [14,19]. PFAS mass balance and transport have been studied for surface water [20,21], groundwater [14,22] and water treatment plants [23,24]. However, there is a certain lack of understanding in interpretation of the retardation factors; for the historical AFFF emission scenarios, the lack of data on both emission rates and periods causes a significant uncertainty in estimates. Field-derived distribution predictors for PFAS are often estimated for different environmental and emission conditions, and show a large variation between sites [25,26,27].

From the environmental perspective, the surfactant nature of PFAS and low concentrations in the carrier medium make it difficult to predict the PFAS transport [27,28]. Laboratory studies have shown that PFAS distribution in aqueous–solid interfaces can be strongly affected by the ionic composition of the aqueous phase [29,30]. The intermolecular interaction between PFAS and solids has been reported as electrostatic and dependent on the solid surface characteristics [29,31]. Furthermore, the molecular chain-length and functional groups have been shown to have an effect on interactions with media [30,32,33]. In the natural heterogeneous media, surfaces of the solids as well as suspended solids, represent a complicated interaction system, including the ionic interactions and impact of the zeta-potential of the system [34,35]. The PFAS interaction with media organic content has been subjected to the hydrophobic interactions and often suggested as a primary driving factor for sorption [29,33]. However, it was also shown that organic matter composition can have a significant impact on PFAS distribution (in connection to PFAS chain-length and functional group) [30].

The scientific data on PFAS in the natural heterogeneous media is still limited and further investigation is required. Understanding of the PFAS behavior in the natural aqueous–solid interfaces is important for the reliability of transport and fate estimates. The detailed investigation is necessary for the verification of the spatial and temporal distribution factors. This is of high importance for far-flied transport prediction and risk assessments.

The objective of the present study was to investigate the vertical PFAS distribution in the sediment cores extracted from the contaminated lake. The specific objectives were: (i) to assess the water and sediment contamination levels; (ii) to evaluate the sediment–water distribution coefficients; and (iii) to investigate the role and impact of the sediment related characteristics on PFAS distribution in the sediment column.

### Study Site

**Figure 1:**
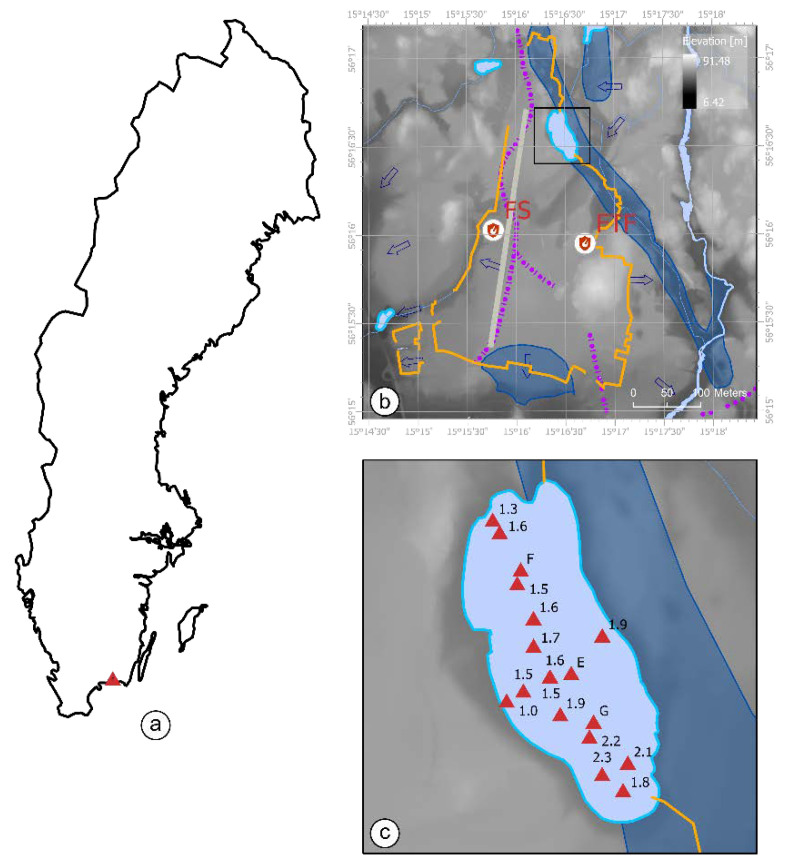
Study site description: (**a**) site location; (**b**) area topography and hydrogeology (including groundwater flow boundaries (purple dot-dash), flow direction (blue arrows) and reservoir (blue area)), airfield territory (solid orange), AFFF emission sources (fire station and fire training facility) and lake location (black box); and (**c**) lake bathymetry and sampling locations. GIS data: GSD Terrängkartan vektor, Lantmäteriet (base map); GSD Höjddata, Lantmäteriet (topography) and SGU Grundvattenmagasin and Grundvatten, SGU (hydrogeology).

The studied Lake Sänksjön is located approximately 100 m northeast from the F17 airfield (Blekinge Air Force Wing) near Ronneby in southern Sweden (Figure 1a). Sänksjön has suggestively been contaminated due to release of the PFAS-containing AFFF at the airfield territory. AFFF was used by the airfield fire brigade for fire training exercises between the 1980s and 2000s (estimated). The fire training activities were mainly conducted for simulation of the aircraft crash and rescue missions (according to former personal). This involved extinguishing of real scale hydrocarbon fuel fires.

There were two main emission sources suggested by Ronneby municipality: fire training facility 900 m south and fire station 1200 m southwest of the Sänksjön (Figure 1b). Due to complex topographic and hydrogeological features of the area, it was difficult to conclude an exact PFAS emission source for the lake. According to the Swedish Geological Survey, the lake body confines an apparent connection to the underlying groundwater (Figure 1b). However, despite PFAS contamination, the interaction with groundwater is unlikely due to groundwater flow direction and lake depth (Figure 1b,c). Therefore, the PFAS transport to Sänksjön was suggestively associated with surface run-off.

## 2. Materials and Methods

### 2.1. Lake Bathymetry and Sampling

Lake water depth was measured using GPS receiver coupled with acoustic sonar on 16 June, 2017; measurements were taken at 18 locations with average water depth of 1.7 ± 0.34 m (Figure 1c).

Duplicate water samples (bulk water) were collected at Locations F and G (corresponding to north and south of the lake, respectively) at 1.6 m (F, *n* = 2) and 2.2 m (G, *n* = 2) water depth using 1 L polypropylene bottles and manual grab sampler on 20–21 June, 2016 (Figure 1c).

Sediment core samples were collected at Locations E–G (corresponding to center, north and south of the lake, respectively) at 1.6 m (E), 1.6 m (F) and 2.2 m (G) depth on 20–21 June 2016. Sediment cores were extracted from the lakebed (in acrylic tube) using manual core sampler. Each sediment core was gently ejected (on site) from the tube (using vertical stand with threaded mechanism), sliced (using acrylic slicer) and transferred into polypropylene jars. Sediment Cores E–G (with 0.34, 0.42 and 0.39 m of depth, respectively), were distributed in segments of 2 cm (*n* = 17, Core E) and 3 cm (*n* = 14, Core F; *n* = 13, Core G).

All samples were stored at 3 °C (water) and −20 °C (sediment) prior to extraction and analysis. All sampling containers (polypropylene bottles, jars, acrylic slicer and core tubes) were pre-rinsed with methanol (×5). Sediment sampler components (slicer and core tubes) were rinsed on site with Milli-Q water (×5) and methanol (×15) prior to each core sampling.

### 2.2. PFAS Target Compounds

In this study, 26 PFAS were analyzed including four perfluoroalkane sulfonates (C_4,6,8,10_ PFSAs) (PFBS, PFHxS, PFOS and PFDS), 13 perfluoroalkyl carboxylates (C_3–13,15,17_ PFCAs) (PFBA, PFPeA, PFHxA, PFHpA, PFOA, PFNA, PFDA, PFUnDA, PFDoDA, PFTriDA, PFTeDA, PFHxDA and PFOcDA), three perfluorooctane sulfonamides (FOSAs) (FOSA, MeFOSA and EtFOSA), two perfluorooctane sulfonamidoethanols (FOSEs) (MeFOSE and EtFOSE), three perfluorooctane sulfonamidoacetic acids (FOSAAs) (FOSAA, MeFOSAA and EtFOSAA) and one fluorotelomer carboxylate (6:2 FTSA). In addition, 16 internal standards (i.e., ^13^C_8_-FOSA, d_3_-MeFOSAA, d_5_-EtFOSAA, d_3_-MeFOSA, d_5_-EtFOSA, d_7_-MeFOSE, d_9_-EtFOSE, ^13^C_4_-PFBA, ^13^C_2_-PFHxA, ^13^C_4_-PFOA, ^13^C_5_-PFNA, ^13^C_2_-PFDA, ^13^C_2_-PFUnDA, ^13^C_2_-PFDoDA, ^18^O_2_-PFHxS and ^13^C_4_-PFOS), and one injection standard (^13^C_8_-PFOA) were used.

### 2.3. PFAS Analysis

The analytical procedures of the sample extraction for water and sediment and PFAS analysis were performed as described elsewhere [36,37,38]. Extracted samples were analyzed using high-performance liquid chromatography coupled to tandem mass-spectrometry (6460 Triple Quadrupole LC/MS System, Agilent Technologies, Santa Clara, CA, USA). Betasil C18 LC column (50 × 2.1 mm, 5 µm particle size, Thermo Fisher Scientific, Waltham, MA, USA) and Hypersil Gold pre-column (10 × 2.1 mm, 5 µm particle size, Thermo Fisher Scientific, Waltham, MA, USA) were used as analytical and guard columns, respectively. The branched isomer concentration of PFHxS and PFOS (i.e., B-PFHxS and B-PFOS) was estimated using the response factors of the respective linear isomers (i.e., L-PFHxS and L-PFOS, respectively).

In total, 4 water and 44 sediment samples were analyzed. Procedural blanks were applied in duplicate for each sediment core batch (*n* = 6) and once for each duplicate water sample (*n* = 4). The method detection limits (MDLs) were determined at an S/N of 3, and ranged 0.04–0.05 ng L^−1^ for water and 0.03–0.4 µg kg^−1^ dry weight (dw) for sediment.

### 2.4. Sediment–Water Partitioning

The field-derived sediment–water partitioning coefficients (*K_d_*) and carbon normalized sediment–water partitioning coefficients (*K_OC_*) were calculated from bulk sediment (ng kg^−1^ dw) and bulk water concentrations (ng L^−1^) as described earlier [36].

### 2.5. Sediment Organic Carbon, Densities

The fraction organic carbon (*f_OC_*) was determined on the replicate sediment core samples by combustion method (disaggregated samples were dried at 105 °C and burned at 1350 °C in a furnace). Sediment bulk and dry bulk densities were determined from direct measurements on wet and dehydrated samples (see Table S1).

### 2.6. Sediment Elemental Analysis

Sediment elemental analysis was performed on the replicate sediment core samples (F and G) using Niton XL3t X-ray fluorescence (XRF) analyser (Thermo Fisher Scientific, Waltham, MA, USA) in soil mode. Samples were prepared and analyzed in compliance with the US EPA method 6200 [39]. Dehydrated (freeze-dried) sediment samples were homogenized, weighed and distributed into XRF sample cups (Premier Lab Supply, Port St. Lucie, FL, USA). In XRF cups, sediment was compressed between Prolene film (Chemplex Industries inc., Palm City, FL, USA) and glass fiber filter (Advantec, Tokyo, Japan). Polyester fiber wool was used as a dumper material (for details see Table S2).

To improve volumetric representation, each sample was scanned two times with X-ray beam collimated at the centre of the sample, and deviating from the center (5–7 mm). To secure repeatability, the XRF instrument was locked in a fixed position and all samples were centered and scanned in an exact manner.

In total, there were twenty-seven sediment samples analyzed. Negative blank and positive reference samples (standard reference material NIST2709a, certified by Rigaku, Tokyo, Japan) were applied for every fifth sample (*n* = 16) and each core sequence (*n* = 2), respectively. Measurements of positive reference samples showed a good agreement with certified concentrations for NIST2709a (see Table S3). The sediment sample concentrations were accordingly adjusted to the averaged negative blank levels.

### 2.7. Statistical Analysis

For measured PFAS concentrations, the relationships with sediment fraction organic carbon were studied using Pearson’s pair-wise correlation (at 95% confidence interval) on data (*n* = 44) including sediment Cores E–G. The relationships between measured sediment elemental content and PFAS concentrations were studied using Pearson correlation, Spearman correlation and Principal Component Analysis (PCA) on standardized data (*n* = 26) including sediment Cores F and G. Related calculations and data evaluation were compiled in MATLAB (MathWorks, Natick, MA USA).

## 3. Results

### 3.1. PFAS in Aqueous Phase

In the Lake Sänksjön water (Locations F and G), 8 of 26 investigated PFAS were detected (i.e., PFHxA, PFHpA, PFOA, PFNA, PFBS, PFHxS, PFOS and 6:2 FTSA) (Tables S4 and S5). The ΣPFAS levels were similar at Locations F and G (95 and 100 ng L^−1^, respectively) and dominated by PFSAs, with a contribution of 84–90% for sum of PFHxS and PFOS (including both linear and branched forms).

### 3.2. PFAS in Sediment

**Figure 2:**
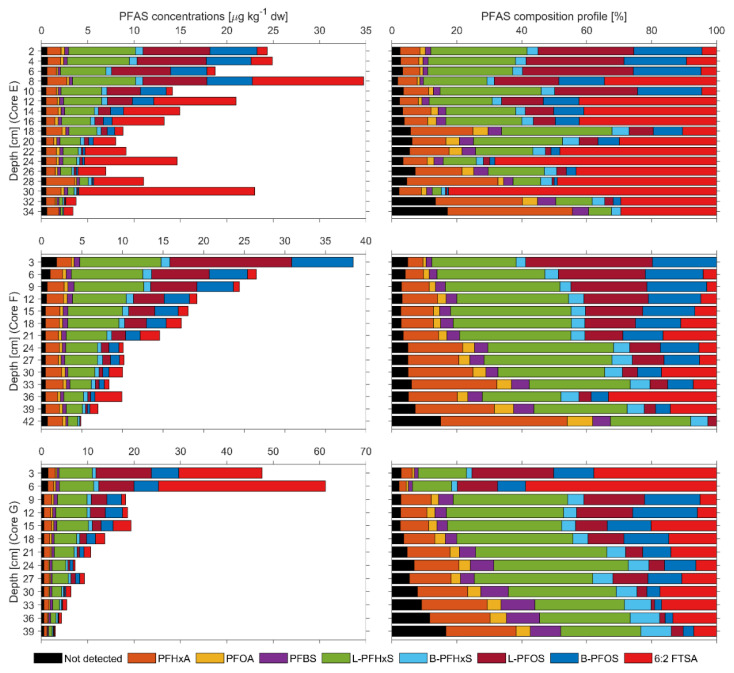
PFAS concentration (µg kg^−1^ dw) and relative composition profile of lake sediment Cores E–G; “not detected” includes concentrations of PFHpA, PFNA, PFDA, PFUnDA, PFDoDa, MeFOSAA, EtFOSAA and 0.5 MDL for the undetected PFAS.

In sediment samples, the ΣPFAS concentration ranged 3.4–25, 4.9–38 and 3–61 µg kg^−1^ dw in the sediment Cores E–G, respectively (Tables S6–S8). The PFAS composition was similar in the sediment cores and dominated by PFHxS and PFOS (sum of linear and branched isomers) with the average contribution (*n* = 44) of 32 ± 11% for PFHxS and 22 ± 16% for PFOS, followed by 6:2 FTSA (21 ± 20%) and PFHxA (14 ± 9%) (Figure 2).

For the remaining PFCAs (PFHpA, PFNA, PFDA, PFUnDA and PFDoDa) and FOSAAs (MeFOSAA and EtFOSAA), the overall contribution was insignificant, as these compounds were inconsistently present in top sediment layers (0–12 cm) with total concentration of 0.6 (E), 2.4 (F) and 2.1 (G) µg kg^−1^ dw.

### 3.3. PFAS Water–Sediment Partitioning

The field-derived sediment–water partitioning coefficient *K_d_* and organic carbon normalized *K_OC_* were calculated for sediment samples from Cores F and G. The sediment–water partitioning coefficients (*K_d_*) ranged 0.6–2.3 L kg^−1^ for individual perfluoroalkyl carboxylates (PFCAs) and 0.9–5.6 L kg^−1^ for individual perfluoroalkane sulfonates (PFSAs). Overall, log *K_d_* and log *K_OC_* values for PFHxA, PFOA, PFBS, PFHxS and PFOS were consistent between F and G, except for 6:2 FTSA (Table S9). For PFSAs, *K_d_* and *K_OC_* values showed an increase with perfluorocarbon moiety as PFBS < B-PFHxS < L-PFOS ≈ B-PFOS, except for L-PFHxS. There was no observed relation with perfluorocarbon chain length for PFCAs.

### 3.4. Sediment Parameters

Sediment samples were identified as *fine detritus gyttia* (Core E) and *coarse detritus gyttia* (Cores F and G). Sediment densities were consistent in sediment Cores E–G with a mean density of 1.0 ± 0.06, 0.97 ± 0.05 and 0.98 ± 0.03 kg L^−1^, respectively. The mean fraction of organic carbon (*f_OC_*) was 0.96 ± 0.03, 0.95 ± 0.04 and 0.95 ± 0.07 for sediment Cores E–G, respectively. The mean dry bulk density (*ρ_dry bulk_*), however, was slightly lower in Core F (0.03 ± 0.01 kg L^−1^) than in Cores E and G (0.04 ± 0.01 and 0.04 ± 0.02 kg L^−1^, respectively). Overall, the sediment cores were similar and considered as a homogeneous media mainly represented by organic matter (Table S1).

Based on XRF analysis, the sediment mineral content in Cores F and G was represented by sulfur (12,000 ± 2900 and 13,000 ± 1900 mg kg^−1^ dw, respectively), iron (9100 ± 1600 and 9100 ± 2300 mg kg^−1^ dw, respectively) and calcium (6800 ± 1500 and 14,000 ± 2800 mg kg^−1^ dw, respectively) (Tables S10 and S11).

### 3.5. PFAS Distribution and Sediment Parameters

The relationship between individual PFAS concentrations and sediment fraction organic carbon, densities and moisture content was studied for four PFCAs (PFHxA, PFHpA, PFOA and PFUnDA), three PFSAs (PFBS, L-PFHxS, B-PFHxS, L-PFOS and B-PFOS), MeFOSAA and 6:2 FTSA (Table S12). Out of eleven studied PFAS, only long-chained PFUnDA (*r* = −0.8, *n* = 8) and L-PFOS (*r* = −0.4, *n* = 43) showed a negative correlation (*p* < 0.05) with fraction organic carbon.

All PFAS and PFOA showed a weak negative correlation with bulk sediment density (Table S12). For the dry bulk density, PFHxA (*r* = −0.4, *n* = 44), PFOA (*r* = −0.8, *n* = 43) and PFBS (*r* = −0.5, *n* = 44) showed a negative correlation. There was no significant correlation observed with moisture content.

The correlation between individual PFAS concentrations and sediment elemental content was studied for PFHxA, PFOA, PFBS, PFBS, L-PFHxS, B-PFHxS, L-PFOS, B-PFOS and 6:2 FTSA (*n* = 26) (Tables S13 and S14). All PFSAs showed a positive correlation (*p* < 0.05) with sulfur (*r_s_* = 0.5–0.6) and titanium (*r_s_* = 0.5–0.6); moreover, for long-chained PFSAs (i.e., PFHxS and PFOS), a positive correlation was found for sediment lead (*r_s_* = 0.6–0.7), arsenic (*r_s_* = 0.6–0.7) and iron (*r_s_* = 0.5–0.6). For PFCAs, PFHxA showed a positive correlation with sediment rubidium, (*r_s_* = 0.4) lead (*r_s_* = 0.6) and arsenic (*r_s_* = 0.6) and a negative correlation with calcium (*r_s_* = −0.5). PFOA was positively correlated with sediment lead (*r_s_* = 0.5), arsenic (*r_s_* = 0.4) and titanium (*r_s_* = 0.4).

**Figure 3:**
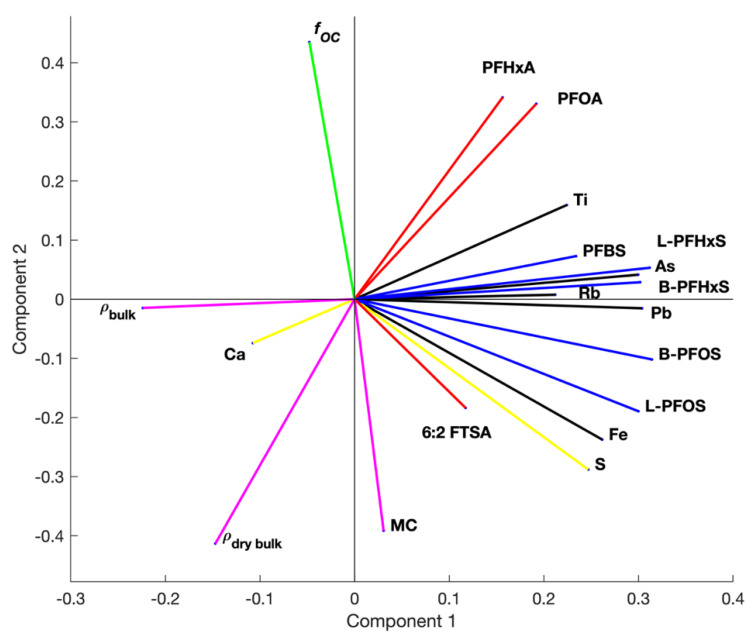
Bi-plot on parameter contribution the data variance within the first (43% explained) and second (24% explained) component spaces, including PFCAs and FTSA (red); PFSAs (blue); sediment iron, lead, rubidium, arsenic and titanium (black); sediment sulfur and calcium (yellow); sediment densities (*ρ_dry bulk_* and *ρ_bulk_*) and moisture content (MC) (magenta); and fraction organic carbon (*f_OC_*, green).

Form the principal component analysis on standardized parametric data (*n* = 26, sediment Cores F and G), the overall data variability was sufficiently explained as 43%, 24% and 12% within the first three component spaces (see Figure S1 for contribution to the variance by each component and parameters). Within the first component space (of 43% explained) (Figure 3), the long-chain PFSAs (L-PFHxS, B-PFHxS, L-PFOS and B-PFOS) followed by sediment arsenic and lead had the significant contribution to the data variability. However, the short-chain PFBS, sediment rubidium and titanium contributions were identical on lower level. L-PFOS, sediment iron and sulfur had a similar contribution to the variance within both the first and second component spaces (with <67% variance explained). PFCAs and contravariant dry bulk density contributions were relevant within first and (on greater level) second component spaces. Contribution of the fraction organic carbon and moisture content was relevant within second component space only and represented by <24% data variability.

Overall, PFSAs variation showed an alignment (correlation) with sediment arsenic, lead, rubidium, titanium and sulfur. PFCAs were negatively correlated to dry bulk density. There was no clear correlation between individual PFASs and fraction organic carbon found.

## 4. Discussion

### 4.1. PFAS in Lake Water and Sediment

The PFAS concentration and composition in water samples from Locations F and G were similar which indicates a spatially uniform emission source and an overall equilibrium of the PFAS masses in the lake water. Considering the lake volume (approximately 12 × 10^4^ m^3^), the total mass of PFAS in the water phase can be estimated as 12 g absolute.

The ΣPFAS concentrations in water detected in the present study were in the same range as reported for PFAS levels in surface water across Sweden [8]. The PFAS composition profile (predominated by PFHxS and PFOS) was similar to reported for sites affected by AFFF release at F18 airfield in Tullinge (suburb of Stockholm, Sweden) [7], Arlanda Stockholm Airport, Sweden [40] and Schiphol Amsterdam Airport, Netherlands [41].

The vertical distribution of PFAS in the sediment column was considered as relatively even and dominated mainly by PFHxS and PFOS. However, the 6:2 FTSA concentration in sediment samples was elevated in the top layer of Core G and ubiquitously present in sediment Core E (corresponding to south and center of the lake, respectively) (Figure 1). This, in agreement with elevated 6:2 FTSA concentration in the corresponding water samples (8.6 ng L^−1^ at Location G) may indicate the recent emission or source located in south of the lake (Figure 1).

There is limited data available for PFAS concentrations in lake sediment and comparison by both concertation and composition is difficult. In the present study, PFAS concentrations (dw) in sediment were similar to those reported for Schiphol Amsterdam Airport, whereas PFHxS concentrations were one order of magnitude higher [41]. The PFAS composition was similar to sediments from Lake Halmsjön [40]. The elevated 6:2 FTSA levels were similar to sediment from Lake Langavatnet [42]. ∑PFAS concentrations in surface sediment (dw) were 1–10 times higher than reported for Laurentian Great Lakes [26].

The vertical distribution data can be very useful for understanding of the sorption and transport processes. However, an exact interpretation of the mass fluxes requires the knowledge on the sediment accumulation rates and temporal boundaries. Unfortunately, the temporal distribution was not possible to determine in present study. There were two independent radioisotope analysis attempts carried out: Pb-210/Ra-226 and Pb-210 analysis (in 2016 and 2017). Due to very low radioisotope signal in sediment, the analysis results were insufficient for an adequate interpretation. The gradient in PFAS concentrations from the bottom to the top of the core indicates both increase in contaminant input (over time) and sorption (retardation) in the vertical transport processes. The MAD (average (mean) absolute deviation) in PFOS and PFHxS concentrations (2.4 and 1.9 (E), 2.8 and 2.3 (F) and 2.5 and 2.1 (G)) was one order of magnitude higher than in PFBS (0.6 (E), 0.12 (F) and 0.1 (G)). This indicates a certain sorption (retardation) in the vertical transport process, with a tendency related to chain the lengths as PFOS > PFHxS > PFBS.

### 4.2. PFAS Water–Sediment Partitioning

In the present study, field-derived log *K_d_* values were slightly higher than reported for corresponding PFAS in lake sediments at Stockholm Arlanda Airport [40] and river sediments at Schiphol Amsterdam Airport [41]. For PFOS, log *K_d_* values were about two times higher than reported for marine sediments [25,43]. It is important to note that PFAS concentrations in sediment can be a result of a recent release or historical emission [7,27,44]. Hence, unless the spatial and temporal conditions are established, field-derived *K_d_* values should be considered with a certain precaution and the local equilibrium conditions have to be considered.

The *K_OC_* values were generally higher than *K_d_* values (Table S9), indicating an affinity of PFAS to organic carbon, which agrees with previous studies [25,26,29]. However, it was suggested to consider the field-derived *K_OC_* values with a certain precaution. Due to the surfactant nature of PFAS and related interaction mechanisms with surfaces, the organic carbon normalized *K_OC_* might not fully represent the media [28,35].

### 4.3. Sediment Composition and PFAS Distribution

The sediment inorganic content was measured in bulk dried samples and can represent both matter of the solids and corresponding metal–ligand complexes in aqueous phase [34]. In the present study, the measured sediment elemental content was primarily subjected to the solid phase due to no significant correlation with sediment dry bulk density and moisture content. Previous studies have shown that sorption of PFAS is impacted by pH and suggested to decrease in *K_d_* values with increasing pH, which is most likely due to pH depending changes in zeta potential of the solid surface [33,45]. It was suggested that the partitioning of PFAS (in particular long-chained PFSAs) in natural media could be affected by the presence of metal oxides and metal-ligand complexes (carbonate, sulfate or phosphate ligands) in aqueous phase. This is however beyond the scope of the present study.

In studied sediments, long-chain PFCAs and PFSAs showed significant correlation with sediment lead, arsenic, iron, titanium and sulfur (*p* < 0.05, Tables S13 and S14). This, in agreement with previous studies, indicates the major effect of the electrostatic interaction with the mineral content of the media [29,31]. The slightly stronger correlation with sediment dry bulk densities (*r_s_* = −0.6–0.7) (Table S13) might also indicate the association with aqueous phase and the mechanical impact of media pore space on PFAS distribution.

The PFAS interaction with the solid organic matter (in dissolved–solid organic matter interface) has been previously reported as hydrophobic [29]. Although, the PFAS sorption on organic porous media can be affected by the organic matter composition [30]. In the present study, derived *K_d_* and *K_OC_* predictors for PFAS, reflected an increased association with organic matter (Table S9). However, further investigation has shown that the organic matter (expressed as *f_OC_*) had no significant effect on PFAS distribution regardless of the functional group or chain-length. Moreover, PFAS distribution was strongly affected by the sediment inorganic content representing <5% of the bulk media. To the best of the authors’ knowledge, this is the first comprehensive study addressing the influence of the sediment inorganic vs. organic content on PFAS distribution in sediment.

Ultimately, the association of PFAS with the solid phase is a complex process that is impacted by the physicochemical properties of PFAS, ionic composition of the aqueous phase, porous media surface charges, structure and composition [30,31,45]. Furthermore, a better understanding is needed in PFAS sorption mechanism, in particular on intermolecular interaction with natural media surfaces and structure.

## 5. Conclusions

PFAS concentrations in the water and sediment core samples were studied for the Lake Sänksjön. In total, eight (out of 26 investigated) different PFAS were detected in the lake water and thirteen different PFAS in the sediment core samples. The PFAS composition in sediment and water was predominated by PFOS and PFHxS; 6:2 FTSA was inconsistently present in water and sediment core samples. The field-derived PFAS partitioning coefficients (log *K_d_* and log *K_oc_*) were identical between compared lake sediment cores (F and G) and showed no apparent trends in relation to perfluorocarbon chain length.

Studied sediment cores were identical in density and high organic matter content. The sediment inorganic content was represented by sulfur, iron, calcium, titanium, lead, arsenic and rubidium. PFAS distribution in sediment was strongly connected to the sediment mineral content (i.e., Fe, Pb, Rb and As). This, in connection to previously reported hydrophobic interaction with media organic matter, indicates the significance of the electrostatic interactions (with media inorganic matter) in sorption processes for PFAS. It was suggested that PFAS sorption on natural heterogeneous media should be addressed with a precaution, in particular for considerations of hydrophobic sorption on media organic matter. Although the overall mass distribution can be connected to the mechanistic retardation processes, the actual physical sorption can be affected by the number of external and media specific parameters. Present findings are of importance for assessment of the PFAS distribution and retardation factors in the natural heterogeneous media as well as for far-field transport estimates.

## Supplementary Materials

The following are available online at https://www.mdpi.com/1660-4601/17/16/5642/s1, Table S1: Densities, fraction organic carbon and moisture content in sediment cores E, F and G respectively, Table S2: Materials description for XRF analysis samples, Table S3: XRF analysis measurement agreement for measured and certified standard, Table S4: PFAS concentrations in water from location F, Table S5: PFAS concentrations in water from location G, Table S6: Individual PFAS concentrations in sediment core E, Table S7: Individual PFAS concentrations in sediment core F, Table S8: Individual PFAS concentrations in sediment core G, Table S9: Sediment-water partitioning (*K_d_*) and organic carbon normalized (*K_OC_*) coefficients in sediment core F and G, Table S10: Elemental composition of the sediment core F, Table S11: Elemental composition of the sediment core G, Table S12: Correlation matrix for Pearson correlation and corresponding p-values on PFAS vs. sediment densities, fraction organic carbon and moisture content, Table S13: Correlation matrix for Spearman’s correlation and corresponding p-values on sediment elemental content vs. PFAS, sediment densities, fraction organic carbon and moisture content, Table S14: Correlation matrix for Pearson correlation and corresponding p-values on sediment elemental content vs. PFAS, sediment densities, fraction organic carbon and moisture content, Figure S1: PCA: variance explained by components and contribution of parameters to component variance.

## References

[1] Ahrens L., Bundschuh M. (2014). Fate and Effects of Poly- and Perfluoroalkyl Substances in the Aquatic Environment: A Review. Environ. Toxicol. Chem..

[2] Buck R.C., Franklin J., Berger U., Conder J.M., Cousins I.T., De Voogt P., Jensen A.A., Kannan K., Mabury S.A., Van Leeuwen S.P. (2011). Perfluoroalkyl and polyfluoroalkyl substances in the environment: Terminology, classification, and origins. Integr. Environ. Assess. Manag..

[3] Ahrens L. (2011). Polyfluoroalkyl compounds in the aquatic environment: A review of their occurrence and fate. J. Environ. Monit..

[4] Armitage J.M., Cousins I.T., Buck R.C., Prevedouros K., Russell M.H., MacLeod M., Korzeniowski S.H. (2006). Modeling global-scale fate and transport of perfluorooctanoate emitted from direct sources. Environ. Sci. Technol..

[5] Cousins I.T., Vestergren R., Wang Z.Y., Scheringer M., McLachlan M.S. (2016). The precautionary principle and chemicals management: The example of perfluoroalkyl acids in groundwater. Environ. Int..

[6] Moody C.A., Field J.A. (1999). Determination of Perfluorocarboxylates in Groundwater Impacted by Fire-Fighting Activity. Environ. Sci. Technol..

[7] Filipovic M., Woldegiorgis A., Norstrom K., Bibi M., Lindberg M., Osteras A.H. (2015). Historical usage of aqueous film forming foam: A case study of the widespread distribution of perfluoroalkyl acids from a military airport to groundwater, lakes, soils and fish. Chemosphere.

[8] Gobelius L., Hedlund J., Durig W., Troger R., Lilja K., Wiberg K., Ahrens L. (2018). Per- and Polyfluoroalkyl Substances in Swedish Groundwater and Surface Water: Implications for Environmental Quality Standards and Drinking Water Guidelines. Environ. Sci. Technol..

[9] Houde M., Martin J.W., Letcher R.J., Solomon K.R., Muir D.C.G. (2006). Biological monitoring of polyfluoroalkyl substances: A review. Environ. Sci. Technol..

[10] Mastrantonio M., Bai E., Uccelli R., Cordiano V., Screpanti A., Crosignani P. (2018). Drinking water contamination from perfluoroalkyl substances (PFAS): An ecological mortality study in the Veneto Region, Italy. Eur. J. Public Health.

[11] Ryota S. (2019). Kyoto University professors detect PFOS concentration of 4 times national average in Ginowan residents’ blood. Ryukyu Shimpo.

[12] Kerger B.D., Copeland T.L., DeCaprio A.P. (2011). Tenuous dose-response correlations for common disease states: Case study of cholesterol and perfluorooctanoate/sulfonate (PFOA/PFOS) in the C8 Health Project. Drug Chem. Toxicol..

[13] Li Y., Fletcher T., Mucs D., Scott K., Lindh C.H., Tallving P., Jakobsson K. (2018). Half-lives of PFOS, PFHxS and PFOA after end of exposure to contaminated drinking water. Occup. Environ. Med..

[14] Shin H.M., Steenland K., Ryan P.B., Vieira V.M., Bartell S.M. (2014). Biomarker-Based Calibration of Retrospective Exposure Predictions of Perfluorooctanoic Acid. Environ. Sci. Technol..

[15] Hansen S., Vestergren R., Herzke D., Melhus M., Evenset A., Hanssen L., Brustad M., Sandanger T.M. (2016). Exposure to per- and polyfluoroalkyl substances through the consumption of fish from lakes affected by aqueous film-forming foam emissions—A combined epidemiological and exposure modeling approach. The SAMINOR 2 Clinical Study. Environ. Int..

[16] Xu Y., Fletcher T., Pineda D., Lindh C.H., Nilsson C., Glynn A., Vogs C., Norstrom K., Lilja K., Jakobsson K. (2020). Serum Half-Lives for Short- and Long-Chain Perfluoroalkyl Acids after Ceasing Exposure from Drinking Water Contaminated by Firefighting Foam. Environ. Health Perspect..

[17] Andersson E.M., Scott K., Xu Y., Li Y., Olsson D.S., Fletcher T., Jakobsson K. (2019). High exposure to perfluorinated compounds in drinking water and thyroid disease. A cohort study from Ronneby, Sweden. Environ. Res..

[18] Li Y., Barregard L., Xu Y., Scott K., Pineda D., Lindh C.H., Jakobsson K., Fletcher T. (2020). Associations between perfluoroalkyl substances and serum lipids in a Swedish adult population with contaminated drinking water. Environ. Health.

[19] Silva A.V., Ringblom J., Lindh C., Scott K., Jakobsson K., Oberg M. (2020). A Probabilistic Approach to Evaluate the Risk of Decreased Total Triiodothyronine Hormone Levels following Chronic Exposure to PFOS and PFHxS via Contaminated Drinking Water. Environ. Health Perspect..

[20] Filipovic M., Laudon H., McLachlan M., Berger U. (2015). Mass Balance of Perfluorinated Alkyl Acids in a Pristine Boreal Catchment. Environ. Sci. Technol..

[21] Moller A., Ahrens L., Surm R., Westerveld J., Van der Wielen F., Ebinghaus R., De Voogt P. (2010). Distribution and sources of polyfluoroalkyl substances (PFAS) in the River Rhine watershed. Environ. Pollut..

[22] Weber A.K., Barber L.B., LeBlanc D.R., Sunderland E.M., Vecitis C.D. (2017). Geochemical and Hydrologic Factors Controlling Subsurface Transport of Poly- and Perfluoroalkyl Substances, Cape Cod, Massachusetts. Environ. Sci. Technol..

[23] Gobelius L., Persson C., Wiberg K., Ahrens L. (2019). Calibration and application of passive sampling for per- and polyfluoroalkyl substances in a drinking water treatment plant. J. Hazard. Mater..

[24] Sinclair E., Kannan K. (2006). Mass loading and fate of perfluoroalkyl surfactants in wastewater treatment plants. Environ. Sci. Technol..

[25] Ahrens L., Yeung L.W.Y., Taniyasu S., Lam P.K.S., Yamashita N. (2011). Partitioning of perfluorooctanoate (PFOA), perfluorooctane sulfonate (PFOS) and perfluorooctane sulfonamide (PFOSA) between water and sediment. Chemosphere.

[26] Remucal C.K. (2019). Spatial and temporal variability of perfluoroalkyl substances in the Laurentian Great Lakes. Environ. Sci. Proc. Imp..

[27] Zareitalabad P., Siemens J., Hamer M., Amelung W. (2013). Perfluorooctanoic acid (PFOA) and perfluorooctanesulfonic acid (PFOS) in surface waters, sediments, soils and wastewater—A review on concentrations and distribution coefficients. Chemosphere.

[28] McCarthy C., Kappleman W., DiGuiseppi W. (2017). Ecological Considerations of Per- and Polyfluoroalkyl Substances (PFAS). Curr. Pollut. Rep..

[29] Jeon J., Kannan K., Lim B.J., An K.G., Kim S.D. (2011). Effects of salinity and organic matter on the partitioning of perfluoroalkyl acid (PFAs) to clay particles. J. Environ. Monit..

[30] Pereira H.C., Ullberg M., Kleja D.B., Gustafsson J.P., Ahrens L. (2018). Sorption of perfluoroalkyl substances (PFASs) to an organic soil horizon - Effect of cation composition and pH. Chemosphere.

[31] Hellsing M.S., Josefsson S., Hughes A.V., Ahrens L. (2016). Sorption of perfluoroalkyl substances to two types of minerals. Chemosphere.

[32] Nouhi S., Ahrens L., Pereira H.C., Hughes A.V., Campana M., Gutfreund P., Palsson G.K., Vorobiev A., Hellsing M.S. (2018). Interactions of perfluoroalkyl substances with a phospholipid bilayer studied by neutron reflectometry. J. Colloid Interface Sci..

[33] Higgins C.P., Luthy R.G. (2006). Sorption of perfluorinated surfactants on sediments. Environ. Sci. Technol..

[34] United States Environmental Protection Agency (1999). Understanding Variation in Partition Coefficient, Kd, Values.

[35] Xiao F. (2015). Comment on “Perfluorooctanoic acid (PFOA) and perfluorooctanesulfonic acid (PFOS) in surface waters, sediments, soils and wastewater—A review on concentrations and distribution coefficients” by Zareitalabad et al. [Chemosphere 91(6) (2013) 725-732]. Chemosphere.

[36] Ahrens L., Yamashita N., Yeung L.W.Y., Taniyasu S., Horii Y., Lam P.K.S., Ebinghaus R. (2009). Partitioning Behavior of Per- and Polyfluoroalkyl Compounds between Pore Water and Sediment in Two Sediment Cores from Tokyo Bay, Japan. Environ. Sci. Technol..

[37] Powley C.R., George S.W., Ryan T.W., Buck R.C. (2005). Matrix effect-free analytical methods for determination of perfluorinated carboxylic acids in environmental matrixes. Anal. Chem..

[38] Taniyasu S., Kannan K., So M.K., Gulkowska A., Sinclair E., Okazawa T., Yamashita N. (2005). Analysis of fluorotelomer alcohols, fluorotelorner acids, and short- and long-chain perfluorinated acids in water and biota. J. Chromatogr. A.

[39] United States Environmental Protection Agency (2007). Field Portable X-ray Fluorescence Spectrometry for the Determination of Elemental Concentrations in Soil and Sediment.

[40] Ahrens L., Norstrom K., Viktor T., Cousins A.P., Josefsson S. (2015). Stockholm Arlanda Airport as a source of per- and polyfluoroalkyl substances to water, sediment and fish. Chemosphere.

[41] Kwadijk C.J.A.F., Kotterman M., Koelmans A.A. (2014). Partitioning of Perfluorooctanesulfonate and Perfluorohexanesulfonate in the Aquatic Environment after an Accidental Release of Aqueous Film Forming Foam at Schiphol Amsterdam Airport. Environ. Toxicol. Chem..

[42] Karrman A., Elgh-Dalgren K., Lafossas C., Moskeland T. (2011). Environmental levels and distribution of structural isomers of perfluoroalkyl acids after aqueous fire-fighting foam (AFFF) contamination. Environ. Chem..

[43] Chen H., Zhang C., Yu Y.X., Han J.B. (2012). Sorption of perfluorooctane sulfonate (PFOS) on marine sediments. Mar. Pollut. Bull..

[44] Awad E., Zhang X.M., Bhavsar S.P., Petro S., Crozier P.W., Reiner E.J., Fletcher R., Tittemier S.A., Braekevelt E. (2011). Long-Term Environmental Fate of Perfluorinated Compounds after Accidental Release at Toronto Airport. Environ. Sci. Technol..

[45] Johnson R.L., Anschutz A.J., Smolen J.M., Simcik M.F., Penn R.L. (2007). The adsorption of perfluorooctane sulfonate onto sand, clay, and iron oxide surfaces. J. Chem. Eng. Data.

