# Supplementary material for: Impact of the Sediment Organic vs. Mineral Content on Distribution of the Per- and Polyfluoroalkyl Substances (PFAS) in Lake Sediment
Source: Int J Environ Res Public Health. 2020 Aug 5;17(16):5642. doi: 10.3390/ijerph17165642 (PMC7459773; doi:10.3390/ijerph17165642)
Supplement: Supplementary file 1 [file ijerph-17-05642-s001.pdf]

Table S1. Densities, fraction organic carbon and moisture content in sediment cores E, F and G respectively

Sediment core	Core depth [cm]	$\rho_{\text{dry bulk}}$ [kg dw L^{-1}]	ρ_{bulk} [kg L^{-1}]	f_{OC}	Moisture
Core E	2	0.08	0.92	0.9	0.83
	4	0.04	1	0.94	0.76
	6	0.05	1	0.94	0.74
	8	0.05	1	0.97	0.59
	10	0.05	1.1	0.97	0.67
	12	0.04	1.1	0.97	0.71
	14	0.05	1.1	0.98	0.68
	16	0.05	1.1	0.98	0.65
	18	0.03	0.96	0.98	0.7
	20	0.04	1	0.99	0.61
	22	0.03	1	0.98	0.81
	24	0.03	1	0.98	0.79
	26	0.03	1	0.98	0.74
	28	0.03	1	0.94	0.86
	30	0.03	1	0.86	0.95
	32	0.06	1	0.96	0.88
	34	0.04	1.1	0.93	0.94
	Mean	0.04	1.02	0.96	0.76
	SD	0.01	0.06	0.03	0.11
Core F	3	0.03	0.93	0.82	0.91
	6	0.03	0.94	0.95	0.73
	9	0.03	0.95	0.95	0.78
	12	0.03	0.92	0.96	0.68
	15	0.03	0.92	0.96	0.71
	18	0.03	0.96	0.97	0.68
	21	0.03	0.94	0.97	0.66
	24	0.03	0.98	0.98	0.61
	27	0.03	1	0.97	0.7
	30	0.03	0.99	0.97	0.66
	33	0.03	1	0.97	0.68
	36	0.04	1	0.98	0.6
	39	0.03	1	0.97	0.76
	42	0.05	1.1	0.92	0.91
	Mean	0.03	0.97	0.95	0.72
	SD	0.01	0.05	0.04	0.09
Core G	3	0.05	0.91	0.91	0.8
	6	0.03	1	0.93	0.87
	9	0.03	1	0.97	0.84
	12	0.03	0.98	0.98	0.75
	15	0.03	0.96	0.98	0.83
	18	0.03	0.99	0.98	0.78
	21	0.03	0.96	0.99	0.73
	24	0.03	0.99	0.99	0.67
	27	0.04	1	0.99	0.71
	30	0.04	0.96	0.99	0.69
	33	0.05	1	0.97	0.79
	36	0.06	0.98	0.9	0.92
	39	0.1	1	0.74	0.9
	Mean	0.04	0.98	0.95	0.79
	SD	0.02	0.03	0.07	0.08

Table S2. Materials description for XRF analysis samples

Material	Producer	Details
Glass Fiber Filter	Advantec, Japan	GA-55, 24mm
Prolene Thin-Film	Chemplex Industries inc., USA	Width 76.2 mm, gauge: 0.00016", 4μm, 40640 Å, typical impurities: Ca, P, Fe, Zn, Cu, Zr, Ti, Al
NIST 2709a	NIST, USA	Standard reference material: San Joaquin Valley soil
XRF Sample Cups	Premier Lab Supply, USA	32mm dia., double open ended
Polyester fiber wool	no information	

Table S3. Measurement agreement between the XRF analysis measured and certified standard (RIGAKU, Japan) values

Measurement	Concentrations [mg kg^{-1} dw]											
	**Zr**	**Sr**	**Pb**	**Zn**	**Fe**	**Mn**	**Cr**	**Ca**	**K**	**Ba**	**Ti**	
certified	150	260	28	140	27.000	510	120	20.000	19.000	810	3500	
measured	140	210	11	98	28.000	470	130	19.000	18.000	820	3400	R^2 = 0.99
measured	140	220	9.2	100	27.000	510	120	20.000	19.000	790	3600	R^2 = 0.99

Table S4. PFAS concentrations in water corresponding to location F

PFAS Concentrations [ng L^{-1}] (Mean of the Duplicate Samples)															
**PFHxA**	**PFHpA**	**PFOA**	**PFNA**	**PFDA**	**PFUnDA**	**PFDoDA**	**PFBS**	**L-PFHxS**	**B-PFHxS**	**L-PFOS**	**B-PFOS**	**MeFOSAA**	**EtFOSAA**	**6:2 FTSA**	**ΣPFAS**
4.8	0.7	2.2	0.15	*	*	*	2.7	45	7.8	13	17	*	*	1.6	95

*, below the Method Detection Limit (MDL)

Table S5. PFAS concentrations in water corresponding to location G

PFAS Concentrations [ng L^{-1}] (Mean of the Duplicate Samples)															
**PFHxA**	**PFHpA**	**PFOA**	**PFNA**	**PFDA**	**PFUnDA**	**PFDoDA**	**PFBS**	**L-PFHxS**	**B-PFHxS**	**L-PFOS**	**B-PFOS**	**MeFOSAA**	**EtFOSAA**	**6:2 FTSA**	**ΣPFAS**
4.9	0.71	2.3	0.19	*	*	*	2.5	45	7.5	13	17	*	*	8.6	100

*, below the Method Detection Limit (MDL)

Table S6. Individual PFAS concentrations in sediment core E.

	**PFAS Concentrations [µg kg^{-1} dw]**															
**Core depth [cm]**	**PFHxA**	**PFHpA**	**PFOA**	**PFNA**	**PFDA**	**PFUnDA**	**PFDoDA**	**PFBS**	**L-PFHxS**	**B-PFHxS**	**L-PFOS**	**B-PFOS**	**MeFOSAA**	**EtFOSAA**	**6:2 FTSA**	**ΣPFAS**
2	1.5	*	0.4	*	*	0.13	*	0.42	7.2	0.83	7.2	5.1	*	*	1.1	24
4	1.4	*	0.31	*	*	0.19	*	0.39	6.7	0.82	7.5	4.8	*	*	2.3	25
6	1	*	0.18	*	*	0.13	*	0.28	4.9	0.57	6.4	3.9	*	*	0.89	19
8	2.1	*	0.27	*	*	0.14	*	0.37	6.8	0.79	6.9	4.9	*	*	12	35
10	1.1	*	0.2	*	*	*	*	0.29	4.4	0.59	3.6	2.8	*	*	0.64	14
12	1.2	*	0.25	*	*	*	*	0.45	4.1	0.6	2.7	2.3	*	*	8.9	21
14	1.3	*	0.32	*	*	*	*	0.37	3.2	0.45	1.3	1.4	*	*	6.1	15
16	0.96	*	0.35	*	*	*	*	0.38	3.1	0.48	0.89	0.97	*	*	5.6	13
18	1.7	*	0.41	*	*	*	*	0.36	3	0.46	0.66	0.79	*	*	0.92	8.8
20	0.83	*	0.34	*	*	*	*	0.34	2.2	0.41	0.46	0.53	*	*	2.4	8
22	1.1	*	0.37	*	*	*	*	0.38	1.6	0.36	0.15	0.25	*	*	4.4	9.1
24	1.1	*	0.3	*	*	*	*	0.41	1.5	0.32	0.29	0.23	*	*	10	15
26	1	*	0.25	*	*	*	*	0.31	1.2	0.26	0.21	0.21	*	*	3	6.9
28	3.1	*	0.19	*	*	*	*	0.32	0.94	0.38	0.082	0.11	*	*	5.4	11
30	1.6	*	0.36	*	*	*	*	0.39	0.65	0.28	0.12	0.11	*	*	19	23
32	1	*	0.17	*	*	*	*	0.21	0.42	0.14	0.1	0.085	*	*	1.1	3.7
34	1.3	*	*	*	*	*	*	0.17	0.24	0.094	*	*	*	*	1	3.4
MDL	<0.08	<0.05	<0.05	<0.05	<0.05	<0.05	<0.05	<0.04	<0.03	<0.03	<0.05	<0.05	<0.05	<0.05	<0.2	

*, below the Method Detection Limit (MDL)

Table S7. Individual PFAS concentrations in sediment core F

Core depth [cm]	PFAS Concentrations [μg kg^{-1} dw]														
	PFHxA	PFHpA	PFOA	PFNA	PFDA	PFUnDA	PFDoDA	PFBS	L-PFHxS	B-PFHxS	L-PFOS	B-PFOS	MeFOSAA	EtFOSAA	6:2 FTSA	ΣPFAS
3	1.8	0.12	0.37	0.11	0.16	0.47	0.12	0.7	10	1.1	15	7.6	0.22	0.14	*	38
6	1.5	0.16	0.46	*	*	0.24	*	0.63	8.8	1.1	7.1	4.7	0.21	0.081	1.1	26
9	2.1	0.17	0.47	*	*	*	*	0.74	8.6	0.84	5.7	4.5	0.092	*	0.74	24
12	2.1	0.13	0.49	*	*	*	*	0.63	6.6	0.91	3.8	3.1	*	*	0.93	19
15	1.8	*	0.36	*	*	*	*	0.61	6.7	0.79	3.2	2.9	*	*	1.2	18
18	1.7	*	0.4	*	*	*	*	0.65	6.3	0.71	2.7	2.4	*	*	1.9	17
21	1.6	*	0.37	*	*	*	*	0.57	5	0.63	1.7	1.8	*	*	2.4	15
24	1.7	*	0.37	*	*	*	*	0.41	3.9	0.5	0.96	1.2	*	*	0.54	10
27	1.6	*	0.34	*	*	*	*	0.45	4	0.64	1	1.1	*	*	0.54	10
30	2	*	0.4	*	*	*	*	0.37	3.3	0.55	0.46	0.74	*	*	1.7	10
33	2.2	*	0.38	*	*	*	*	0.45	2.6	0.51	0.45	0.66	*	*	0.6	8.4
36	1.5	*	0.32	*	*	*	*	0.44	2.4	0.55	0.38	0.52	*	*	3.3	9.9
39	1.7	*	0.41	*	*	*	*	0.44	2	0.37	0.24	0.32	*	*	0.99	7
42	1.9	*	0.38	*	*	*	*	0.27	1.2	0.26	0.13	*	*	*	*	4.9
MDL	<0.08	<0.05	<0.05	<0.05	<0.05	<0.05	<0.05	<0.04	<0.03	<0.03	<0.05	<0.05	<0.05	<0.05	<0.2	

*, below the Method Detection Limit (MDL)

Table S8. Individual PFAS concentrations in sediment core G

Core depth [cm]	PFAS Concentrations in Sediment [μg kg^{-1} dw]														
	PFHxA	PFHpA	PFOA	PFNA	PFDA	PFUnDA	PFDoDA	PFBS	L-PFHxS	B-PFHxS	L-PFOS	B-PFOS	MeFOSAA	EtFOSAA	6:2 FTSA	ΣPFAS
3	1.6	0.047	0.31	0.11	0.21	0.33	0.092	0.54	7.1	0.78	12	5.9	0.18	0.083	18	48
6	1.3	0.26	0.44	0.087	0.13	0.28	*	0.74	7.4	1.1	7.6	5.3	0.24	*	36	61
9	1.7	*	0.41	*	*	*	*	0.82	6.4	0.91	3.4	3.1	*	*	0.93	18
12	1.5	*	0.46	*	*	*	*	0.66	6.7	0.74	3.2	3.7	*	*	1.1	19
15	1.7	*	0.48	*	*	*	*	0.64	6.8	0.82	1.9	2.6	*	*	3.9	19
18	1.3	*	0.44	*	*	*	*	0.48	4.9	0.63	1.5	1.9	*	*	2	14
21	1.4	*	0.31	*	*	*	*	0.54	4.3	0.61	0.56	0.92	*	*	1.5	11
24	1	*	0.25	*	*	*	*	0.53	3	0.46	0.35	0.7	*	*	0.46	7.3
27	1.2	*	0.26	*	*	*	*	0.4	3.4	0.57	1	0.97	*	*	1	9.3
30	0.98	*	0.25	*	*	*	*	0.54	2.1	0.41	0.19	0.26	*	*	1.1	6.3
33	1.1	*	0.23	*	*	*	*	0.57	1.5	0.45	0.058	0.12	*	*	0.92	5.4
36	0.8	*	0.22	*	*	*	*	0.44	1.2	0.39	0.074	0.1	*	*	0.58	4.3
39	0.65	*	0.13	*	*	*	*	0.28	0.74	0.28	0.11	0.099	*	*	0.21	3
MDL	<0.08	<0.05	<0.05	<0.05	<0.05	<0.05	<0.05	<0.04	<0.03	<0.03	<0.05	<0.05	<0.05	<0.05	<0.2	

*, below the Method Detection Limit (MDL)

Table S9. Sediment-water partitioning (*K_d*)[a] and organic carbon normalized (*K_{OC}*)[b] coefficients in sediment core F and G.

Distribution Predictor	Sediment Core	PFHxA	PFOA	PFBS	L-PFHxS	B-PFHxS	L-PFOS	B-PFOS	6:2 FTSA
Log *K_d* [L kg^{-1}]	F (*n* = 14)	2.3 ± 0.1	0.6 ± 0.1	1.0 ± 0.1	5.6 ± 0.3	2.5 ± 0.2	3.5 ± 0.6	3.9 ± 0.4 (*n* = 13)	0.7 ± 0.3 (*n* = 12)
	G (*n* = 13)	2.2 ± 0.1	0.6 ± 0.1	0.9 ± 0.1	5.5 ± 0.3	2.4 ± 0.2	3.2 ± 0.8	3.8 ± 0.7	3.0 ± 0.6
Log *K_{OC}* [L kg^{-1}]	F (*n* = 14)	4.3 ± 0.1	2.6 ± 0.1	3.0 ± 0.1	7.6 ± 0.3	4.5 ± 0.2	5.5 ± 0.6	5.9 ± 0.5 (*n* = 13)	2.7 ± 0.2 (*n* = 12)
	G (*n* = 13)	4.2 ± 0.1	2.6 ± 0.2	2.9 ± 0.1	7.5 ± 0.3	4.4 ± 0.2	5.3 ± 0.8	5.7 ± 0.7	5.0 ± 0.6

[a] $K_d = C_{sediment}/C_{dissolved\ phase}$; [b] $K_{OC} = K_d \times 100/f_{OC}$

Table S10. Elemental composition of the sediment core F

Core Depth [cm]	Concentrations [mg kg^{-1} dw]						
	Rb	Pb	As	Fe	Ti	Ca	S
6	45	310	76	11.000	1700	6300	18.000
	45	320	70	11.000	1700	6000	18.000
3	38	270	64	12.000	1300	8400	18.000
	35	270	64	13.000	*	7700	18.000
9	45	200	62	10.000	1800	5500	14.000
	44	210	62	11.000	1600	4000	13.000
12	43	160	47	11.000	1700	6400	13.000
	36	120	48	9300	1500	5800	12.000
15	corrupted sample						
18	38	120	37	9400	1500	8400	12.000
	36	120	39	9300	1500	7800	12.000
21	35	81	38	8500	1300	8500	11.000
	34	82	32	8600	1300	8000	10.000
24	32	65	28	8300	*	9300	10.000
	33	67	27	8400	*	9000	10.000
27	31	74	22	8900	*	9000	11.000
	30	71	30	8800	*	8200	10.000
30	31	67	20	8200	*	8600	10.000
	31	68	23	8200	*	8300	10.000
33	29	66	22	8200	*	8700	9900
	32	68	22	7900	*	8000	9900
36	27	65	23	7100	*	9500	10.000
	29	68	24	7500	*	8900	10.000
39	23	49	21	7500	*	10.000	10.000
	23	55	18	7400	*	9400	9800
42	25	41	14	7800	*	10.000	10.000
	22	39	15	8000	*	9700	10.000

*, not detected

Table S11. Elemental composition of the sediment core G.

Core Depth [cm]	Concentrations [mg kg^{-1} dw]						
	Rb	Pb	As	Fe	Ti	Ca	S
3	34	210	57	15.000	*	14.000	17.000
	33	210	55	15.000	*	13.000	19.000
6	25	92	29	13.000	*	17.000	17.000
	27	83	29	13.000	*	14.000	15.000
9	16	27	13	9200	*	18.000	15.000
	15	23	12	9200	*	15.000	13.000
12	14	10	*	7100	*	18.000	12.000
	15	15	*	7100	*	17.000	12.000
15	15	*	*	7000	*	18.000	11.000
	14	*	*	6600	*	17.000	11.000
18	21	15	*	7700	*	17.000	13.000
	21	13	*	7800	*	16.000	12.000
21	18	*	*	7200	*	16.000	13.000
	18	*	*	7400	*	15.000	12.000
24	17	*	*	8500	*	16.000	13.000
	17	*	*	9000	*	16.000	13.000
27	17	*	*	9000	*	15.000	14.000
	17	*	*	9100	*	14.000	13.000
30	16	*	*	8500	*	13.000	12.000
	16	*	*	9000	*	12.000	12.000
33	20	*	*	8700	*	13.000	14.000
	21	*	*	8800	*	11.000	13.000
36	20	*	*	8500	*	11.000	13.000
	21	*	*	8700	*	10.000	12.000
39	32	*	*	7300	*	8900	13.000
	39	*	*	7800	*	8800	12.000

*, not detected

Table S12. Correlation matrix for Pearson correlation (pairwise) and corresponding *p*-values (in grey, α = 0.05) on PFAS vs sediment densities, fraction organic carbon and moisture content (on data including sediment cores E, F and G).

Parameter	n	PFHxA	PFHpA	PFOA	PFNA	PFDA	PFUnDA	PFBS	L-PFHxS	B-PFHxS	L-PFOS	B-PFOS	MeFOSAA	EtFOSAA	6:2 FTSA	$\rho_{dry\ bulk}$	ρ_{bulk}	f_{OC}	Moisture
PFHxA *	44		−0.33	0.41	0.92	0.48	0.26	0.24	0.3	0.31	0.18	0.25	−0.84	0.95	0.039	−0.43	−0.26	0.054	−0.04
			0.52	0.006	0.26	0.68	0.54	0.12	0.045	0.038	0.25	0.11	0.075	0.19	0.81	0.0039	0.094	0.73	0.81
PFHpA *	6	−0.33		0.62	−0.94	−0.95	−0.4	0.8	0.035	0.58	−0.43	−0.21	0.22	0.14	0.4	−0.68	0.95	0.29	0.19
		0.52		0.19	0.22	0.21	0.6	0.055	0.95	0.22	0.39	0.69	0.73	0.91	0.5	0.14	0.0035	0.58	0.72
PFOA *	43	0.41	0.62		−0.89	−0.98	0.36	0.61	0.55	0.55	0.21	0.36	−0.23	−0.14	0.098	−0.57	−0.33	0.21	−0.07
		0.0061	0.19		0.31	0.12	0.39	1.7E−05	0.00014	0.0001	0.18	0.02	0.71	0.91	0.54	7.8E−05	0.028	0.17	0.64
PFNA *	3	0.92	−0.94	−0.89		0.79	0.7	−0.65	0.42	−0.5	0.92	0.7	−0.76	0	−1	0.64	−0.98	−0.64	−0.16
		0.26	0.22	0.31		0.42	0.5	0.55	0.73	0.67	0.26	0.5	0.45	0	0	0.55	0.14	0.56	0.9
PFDA *	3	0.48	−0.95	−0.98	0.79		0.11	−0.98	−0.24	−0.93	0.47	0.11	−1	−1	−1	0.98	−0.9	−0.028	−0.73
		0.68	0.21	0.12	0.42		0.93	0.12	0.85	0.24	0.69	0.93	0.03	0	0	0.13	0.29	0.98	0.48
PFUnDA *	8	0.26	−0.4	0.36	0.7	0.11		0.79	0.77	0.63	0.92	0.9	−0.013	0.93	0.63	−0.51	−0.46	−0.77	0.66
		0.54	0.6	0.39	0.5	0.93		0.02	0.027	0.095	0.0013	0.0022	0.99	0.23	0.13	0.2	0.25	0.026	0.08
PFBS *	44	0.24	0.8	0.61	−0.65	−0.98	0.79		0.71	0.77	0.41	0.5	−0.099	0.81	0.17	−0.48	−0.55	0.082	−0.03
		0.12	0.055	2E−05	0.55	0.12	0.02		6.3E−08	1E−09	0.0063	0.00079	0.87	0.4	0.29	0.001	0.0001	0.6	0.84
L-PFHxS *	44	0.3	0.035	0.55	0.42	−0.24	0.77	0.71		0.96	0.83	0.93	−0.0028	0.79	0.15	−0.28	−0.5	−0.031	−0.15
		0.045	0.95	1E−04	0.73	0.85	0.027	6.3E−08		7E−24	1E−11	1.9E−18	1	0.42	0.35	0.067	0.0006	0.84	0.34
B-PFHxS *	44	0.31	0.58	0.55	−0.5	−0.93	0.63	0.77	0.96		0.78	0.87	0.75	0.47	0.23	−0.3	−0.49	−0.026	−0.15
		0.038	0.22	1E-04	0.67	0.24	0.095	9.9E−10	6.9E−24		9.8E−10	4.6E−14	0.15	0.69	0.14	0.05	0.0006	0.87	0.34

L-PFOS *	43	0.18	−0.43	0.21	0.92	0.47	0.92	0.41	0.83	0.78		0.96	0.41	0.81	0.39	−0.01	−0.37	−0.35	0.15
		0.25	0.39	0.18	0.26	0.69	0.0013	0.0063	1E−11	1E−09		3.5E−24	0.49	0.4	0.013	0.95	0.014	0.02	0.34
B-PFOS *	42	0.25	−0.21	0.36	0.7	0.11	0.9	0.5	0.93	0.87	0.96		0.46	0.92	0.32	−0.072	−0.35	−0.26	0.11
		0.11	0.69	0.02	0.5	0.93	0.0022	0.00079	1.9E−18	5E−14	3.5E−24		0.44	0.25	0.042	0.65	0.024	0.1	0.49
MeFOSAA *	5	−0.84	0.22	−0.23	−0.76	−1	−0.013	−0.099	−0.0028	0.75	0.41	0.46		0.67	0.65	0.21	0.26	−0.38	0.45
		0.075	0.73	0.71	0.45	0.03	0.99	0.87	1	0.15	0.49	0.44		0.53	0.35	0.74	0.67	0.52	0.45
EtFOSAA *	3	0.95	0.14	−0.14	0	−1	0.93	0.81	0.79	0.47	0.81	0.92	0.67		1	−0.36	0.16	−0.96	0.93
		0.19	0.91	0.91	0	0	0.23	0.4	0.42	0.69	0.4	0.25	0.53		0	0.76	0.9	0.18	0.23
6:2 FTSA *	42	0.039	0.4	0.098	−1	−1	0.63	0.17	0.15	0.23	0.39	0.32	0.65	1		−0.089	0.061	−0.19	0.25
		0.81	0.5	0.54	0	0	0.13	0.29	0.35	0.14	0.013	0.042	0.35	0		0.57	0.7	0.23	0.11

*, significant correlations marked as red text

Table S13. Correlation matrix for Spearman correlation and corresponding *p*-values (in grey, α=0.05) on sediment elemental content vs PFAS, sediment densities, fraction organic carbon and moisture content (on data including sediment cores F and G, $n = 26$).

Parameter	Rb	Pb	As	Fe	Ti	Ca	S	PFHxA	PFOA	PFBS	L-PFHxS	B-PFHxS	L-PFOS	B-PFOS	6:2 FTSA	$\rho_{dry\ bulk}$	ρ_{bulk}	f_{OC}	Moisture
Rb*		0.81	0.84	0.47	0.73	-0.89	0.11	0.41	0.18	0.041	0.3	0.28	0.37	0.31	-0.12	-0.04	-0.4	-0.61	-0.16
		6.1E−07	1.1E−07	0.016	3E−05	1E−09	0.58	0.038	0.37	0.84	0.13	0.17	0.066	0.13	0.57	0.84	0.043	0.0011	0.43
Pb*	0.81		0.98	0.61	0.68	−0.69	0.22	0.61	0.49	0.36	0.64	0.62	0.71	0.66	0.14	−0.35	−0.44	−0.51	−0.13
	6.1E−07		3.3E−17	0.001	0.0001	9E−05	0.29	0.001	0.012	0.072	0.00045	0.00066	5.1E−05	0.00028	0.51	0.077	0.025	0.0073	0.52
As*	0.84	0.98		0.63	0.71	−0.75	0.22	0.59	0.4	0.34	0.6	0.58	0.66	0.61	0.074	−0.31	−0.46	−0.51	−0.16
	1.1E−07	3.3E−17		0.00059	4E−05	1E−05	0.28	0.0015	0.042	0.089	0.0013	0.0021	0.00025	0.0011	0.72	0.12	0.019	0.0082	0.43
Fe*	0.47	0.61	0.63		0.51	−0.4	0.71	0.098	0.045	0.45	0.5	0.54	0.57	0.55	−0.04	−0.037	−0.37	−0.43	0.13
	0.016	0.001	0.00059		0.0078	0.045	0.00005	0.64	0.83	0.021	0.0089	0.0047	0.0024	0.0038	0.85	0.86	0.06	0.027	0.52
Ti*	0.73	0.68	0.71	0.51		−0.74	0.32	0.39	0.43	0.5	0.56	0.54	0.54	0.5	−0.0037	−0.45	−0.64	−0.34	−0.13
	2.7E−05	0.00014	4.2E−05	0.0078		2E−05	0.11	0.05	0.027	0.0098	0.0027	0.0044	0.004	0.0088	0.99	0.021	0.0004	0.088	0.53
Ca*	−0.89	−0.69	−0.75	−0.4	−0.74		0.065	−0.5	−0.14	0.0072	−0.11	−0.13	−0.16	−0.088	0.28	0.063	0.29	0.5	0.27
	1.2E−09	8.8E−05	1.1E−05	0.045	2E−05		0.75	0.01	0.51	0.97	0.58	0.53	0.44	0.67	0.17	0.76	0.15	0.0096	0.18
S*	0.11	0.22	0.22	0.71	0.32	0.065		−0.25	0.012	0.58	0.56	0.58	0.54	0.57	0.028	−0.065	−0.41	−0.32	0.48
	0.58	0.29	0.28	0.00005	0.11	0.75		0.22	0.95	0.0018	0.0031	0.0019	0.0043	0.0023	0.89	0.75	0.039	0.11	0.01
PFHxA*	0.41	0.61	0.59	0.098	0.39	−0.5	−0.25		0.63	0.17	0.35	0.32	0.39	0.33	−0.086	−0.5	−0.17	−0.22	−0.17
	0.038	0.001	0.0015	0.64	0.05	0.01	0.22		0.0006	0.41	0.078	0.11	0.049	0.1	0.68	0.0096	0.4	0.29	0.42
PFOA*	0.18	0.49	0.4	0.045	0.43	−0.14	0.012	0.63		0.51	0.64	0.63	0.62	0.6	0.31	−0.81	−0.23	−0.063	−0.01
	0.37	0.012	0.042	0.83	0.027	0.51	0.95	0.00059		0.0084	0.00043	0.00058	0.00071	0.0011	0.13	4.8E−07	0.25	0.76	0.96

PFBS*	0.041	0.36	0.34	0.45	0.5	0.0072	0.58	0.17	0.51		0.78	0.8	0.71	0.75	0.29	−0.71	−0.48	−0.086	0.19
	0.84	0.072	0.089	0.021	0.0098	0.97	0.0018	0.41	0.0084		2.4E−06	1E−06	4.4E−05	0.00001	0.15	4.2E−05	0.014	0.68	0.34
L-PFHxS*	0.3	0.64	0.6	0.5	0.56	−0.11	0.56	0.35	0.64	0.78		0.96	0.97	0.98	0.37	−0.7	−0.63	−0.13	0.07
	0.13	0.00045	0.0013	0.0089	0.0027	0.58	0.0031	0.078	0.0004	2.4E−06		1.5E−14	1.6E−16	6E−19	0.063	7.8E−05	0.0006	0.51	0.72
B-PFHxS*	0.28	0.62	0.58	0.54	0.54	-0.13	0.58	0.32	0.63	0.8	0.96		0.95	0.95	0.38	-0.7	-0.52	-0.17	0.08
	0.17	0.00066	0.0021	0.0047	0.0044	0.53	0.0019	0.11	0.0006	1E−06	1.5E−14		1.5E−13	2.9E−13	0.057	6.5E−05	0.0069	0.41	0.68
L-PFOS*	0.37	0.71	0.66	0.57	0.54	−0.16	0.54	0.39	0.62	0.71	0.97	0.95		0.99	0.36	−0.64	−0.57	−0.22	0.08
	0.066	5.1E−05	0.00025	0.0024	0.004	0.44	0.0043	0.049	0.0007	4.4E−05	1.6E−16	1.5E−13		6.3E−20	0.072	0.00043	0.0023	0.29	0.71
B-PFOS*	0.31	0.66	0.61	0.55	0.5	−0.088	0.57	0.33	0.6	0.75	0.98	0.95	0.99		0.36	−0.64	−0.59	−0.17	0.08
	0.13	0.00028	0.0011	0.0038	0.0088	0.67	0.0023	0.1	0.0011	0.00001	6E−19	2.9E−13	6.3E−20		0.068	0.00047	0.0014	0.42	0.71
6:2 FTSA*	−0.12	0.14	0.074	−0.04	−0.0037	0.28	0.028	−0.086	0.31	0.29	0.37	0.38	0.36	0.36		−0.35	−0.25	0.26	−0.21
	0.57	0.51	0.72	0.85	0.99	0.17	0.89	0.68	0.13	0.15	0.063	0.057	0.072	0.068		0.081	0.21	0.19	0.31

*, significant correlations marked as red text

Table S14. Correlation matrix for Pearson correlation and corresponding *p*-values (in grey, α=0.05) on sediment elemental content vs PFAS, sediment densities, fraction organic carbon and moisture content (on data including sediment cores F and G, *n* = 26).

Parameter	Rb	Pb	As	Fe	Ti	Ca	S	PFHxA	PFOA	PFBS	L-PFHxS	B-PFHxS	L-PFOS	B-PFOS	6:2 FTSA	$\rho_{dry\ bulk}$	ρ_{bulk}	f_{OC}	Moisture
Rb *		0.8	0.86	0.43	0.74	−0.86	0.27	0.41	0.23	0.06	0.4	0.34	0.44	0.41	0.015	0.045	−0.41	−0.38	−0.13
		7.2E−07	2.5E−08	0.027	1.4E−05	2.6E−08	0.18	0.039	0.25	0.77	0.043	0.086	0.024	0.04	0.94	0.83	0.04	0.054	0.54
Pb *	0.8		0.97	0.72	0.68	−0.59	0.65	0.46	0.42	0.38	0.72	0.67	0.8	0.76	0.15	−0.25	−0.55	−0.25	0.02
	7.2E−07		7.6E−17	0.00004	0.00013	0.0015	0.00036	0.017	0.035	0.054	3.1E−05	0.0002	9E−07	6.2E−06	0.46	0.21	0.0037	0.22	0.92
As *	0.86	0.97		0.69	0.73	−0.67	0.53	0.55	0.46	0.38	0.69	0.64	0.73	0.71	0.17	−0.3	−0.54	−0.19	−0.07
	2.5E−08	7.6E−17		0.00011	2.3E−05	0.00017	0.0053	0.0033	0.019	0.054	8.9E−05	0.0004	2E−05	4.7E−05	0.39	0.14	0.0048	0.36	0.74
Fe *	0.43	0.72	0.69		0.28	−0.17	0.84	0.11	0.097	0.43	0.6	0.63	0.88	0.78	0.6	−0.074	−0.48	−0.31	0.26
	0.027	0.00004	0.00011		0.17	0.4	7.4E−08	0.6	0.64	0.028	0.0012	0.0006	5E−09	2.1E−06	0.0011	0.72	0.013	0.13	0.19
Ti *	0.74	0.68	0.73	0.28		−0.62	0.29	0.38	0.44	0.44	0.57	0.49	0.34	0.42	−0.14	−0.29	−0.54	−0.015	−0.17
	1.4E−05	0.00013	2.3E−05	0.17		0.00072	0.16	0.055	0.024	0.023	0.0025	0.01	0.088	0.031	0.51	0.15	0.0046	0.94	0.4
Ca *	−0.86	−0.59	−0.67	−0.17	−0.62		0.037	−0.4	−0.063	0.15	−0.034	−0.0074	−0.12	−0.036	0.25	−0.1	0.17	0.28	0.2
	2.6E−08	0.0015	0.00017	0.4	0.00072		0.86	0.043	0.76	0.45	0.87	0.97	0.56	0.86	0.22	0.62	0.41	0.17	0.33
S *	0.27	0.65	0.53	0.84	0.29	0.037		−0.13	0.049	0.55	0.66	0.69	0.84	0.79	0.41	−0.011	−0.51	−0.34	0.42
	0.18	0.00036	0.0053	7.4E−08	0.16	0.86		0.54	0.81	0.0037	0.00023	8E−05	7E−08	1.3E−06	0.038	0.96	0.0074	0.085	0.03
PFHxA *	0.41	0.46	0.55	0.11	0.38	−0.4	−0.13		0.75	0.22	0.43	0.36	0.25	0.32	−0.077	−0.62	−0.11	0.26	−0.24
	0.039	0.017	0.0033	0.6	0.055	0.043	0.54		1E−05	0.27	0.029	0.075	0.21	0.11	0.71	0.00075	0.61	0.19	0.23

PFOA *	0.23	0.42	0.46	0.097	0.44	−0.063	0.049	0.75		0.52	0.67	0.63	0.31	0.5	0.15	−0.77	−0.19	0.38	−0.11
	0.25	0.035	0.019	0.64	0.024	0.76	0.81	9.9E−06		0.0069	0.00018	0.0006	0.12	0.0091	0.47	4E−06	0.36	0.058	0.58
PFBS *	0.06	0.38	0.38	0.43	0.44	0.15	0.55	0.22	0.52		0.79	0.81	0.56	0.71	0.29	−0.58	−0.52	0.18	0.11
	0.77	0.054	0.054	0.028	0.023	0.45	0.0037	0.27	0.0069		1.4E−06	5E−07	0.0031	0.00005	0.15	0.0018	0.0061	0.38	0.6
L-PFHxS *	0.4	0.72	0.69	0.6	0.57	−0.034	0.66	0.43	0.67	0.79		0.94	0.81	0.93	0.3	−0.56	−0.63	−0.011	0.1
	0.043	3.1E−05	8.9−05	0.0012	0.0025	0.87	0.00023	0.029	0.0002	1.4E−06		6E−13	4E−07	3.4E−12	0.14	0.0029	0.0006	0.96	0.64
B-PFHxS *	0.34	0.67	0.64	0.63	0.49	−0.0074	0.69	0.36	0.63	0.81	0.94		0.78	0.89	0.41	−0.55	−0.57	0.0041	0.11
	0.086	0.00017	0.0004	0.00062	0.01	0.97	8.2E−05	0.075	0.0006	4.5E−07	6.2E−13		3E−06	9.7E−10	0.036	0.0039	0.0026	0.98	0.6
L-PFOS *	0.44	0.8	0.73	0.88	0.34	−0.12	0.84	0.25	0.31	0.56	0.81	0.78		0.96	0.43	−0.2	−0.56	−0.38	0.34
	0.024	8.8E−07	1.9E−05	4.5E−09	0.088	0.56	7.1E−08	0.21	0.12	0.0031	4.1E−07	3E−06		2.7E−14	0.028	0.34	0.0029	0.058	0.09
B-PFOS *	0.41	0.76	0.71	0.78	0.42	−0.036	0.79	0.32	0.5	0.71	0.93	0.89	0.96		0.44	−0.36	−0.58	−0.24	0.28
	0.04	6.2E−06	4.7E−05	2.1E−06	0.031	0.86	1.3E−06	0.11	0.0091	0.00005	3.4E−12	1E−09	3E−14		0.023	0.074	0.0017	0.24	0.16
6:2 FTSA *	0.015	0.15	0.17	0.6	−0.14	0.25	0.41	−0.077	0.15	0.29	0.3	0.41	0.43	0.44		−0.074	−0.084	−0.077	0.23
	0.94	0.46	0.39	0.0011	0.51	0.22	0.038	0.71	0.47	0.15	0.14	0.036	0.028	0.023		0.72	0.68	0.71	0.27

*, significant correlations marked as red text

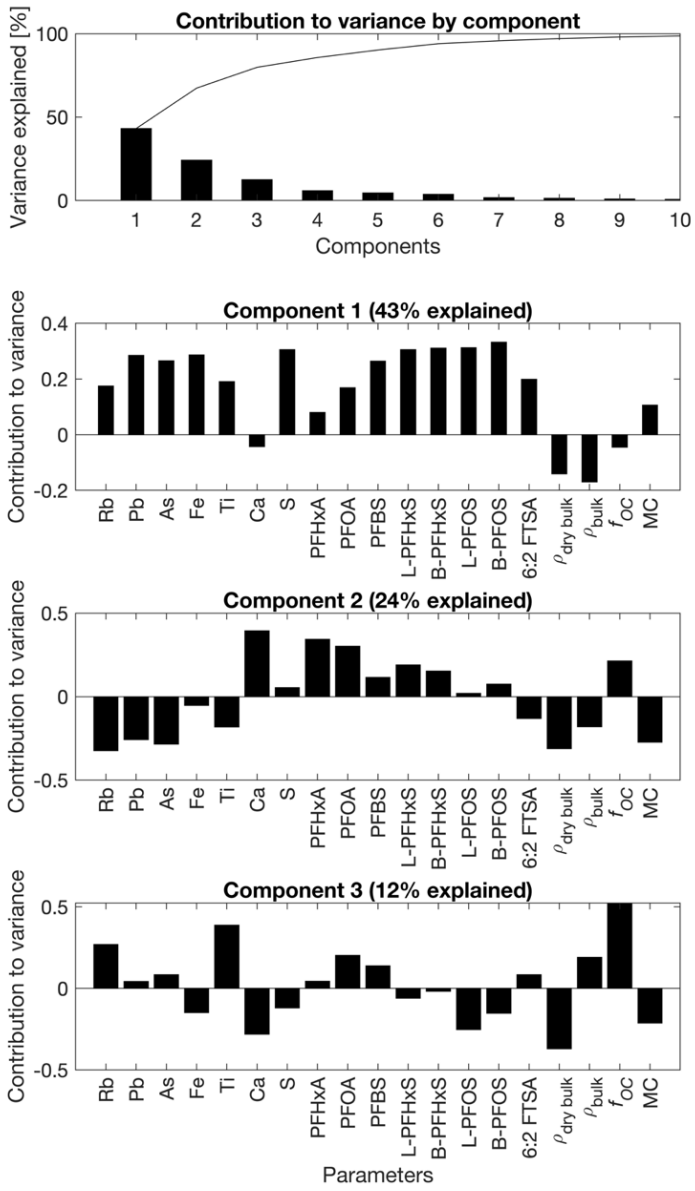

Figure S1. PCA data from top to bottom: overall variance explained by components, contribution of parameters to variance of the component 1, 2 and 3 respectively.
